# Supplementary material for: Lab-on-device investigation of phase transition in MoOx semiconductors
Source: Nat Commun. 2025 May 23;16:4784. doi: 10.1038/s41467-025-60050-7 (PMC12098734; doi:10.1038/s41467-025-60050-7)
Supplement: Supplementary file 1 — Supplementary Information [file 41467_2025_60050_MOESM1_ESM.pdf]

Supplementary Information

Lab-on-device investigation of phase transition in MoO_x semiconductors

Xiaoci Liang[1], Dongyue Su[1], Younian Tang[2], Bin Xi[2], Chunzhen Yang[3], Huixin Xiu[4], Jialiang Wang[5], Chuan Liu[1*], Mengye Wang[3*], Yang Chai[5*]

[1]State Key Laboratory of Optoelectronic Materials and Technologies and the Guangdong Province Key Laboratory of Display Material and Technology, School of Electronics and Information technology, Sun Yat-Sen University, Guangzhou, China
[2]School of Materials Science and Engineering, Sun Yat-sen University, Guangzhou, China
[3] School of Materials, Sun Yat-Sen University, Shenzhen, China
[4]School of Materials Science and Engineering, University of Shanghai for Science and Technology, Shanghai, China
[5]Department of Applied Physics, The Hong Kong Polytechnic University, Hung Hom, Kowloon, Hong Kong, China

Table of content

Supplementary Note 1: MoO_x ECRAM performance and film characterization

Supplementary Note 2: DFT calculation.

Supplementary Note 3: The integration of the MoO_x ECRAM

Supplementary Note 4: The hardware spiking neural network

Supplementary Reference

Supplementary Note 1: MoO_x ECRAM performance and film characterization

The transport of the proton from Nafion into MoO_x, there are diffusion barrier in the bulk of Nafion and MoO_x and the interface between Nafion into MoO_x (**Figure S3a**). Due to the interfacial potential barrier, at low gate voltages or currents, the driving force for proton injection is insufficient to overcome this barrier, resulting in low proton flux (Q_H) and minimal conductance changes. The barrier, influenced by factors like work functions and defects, can be overcome by increasing the gate voltage or current, which enhances proton injection and flux. This relationship is described by the Poisson equation, linking charge accumulation to potential changes. Thus, proton flux is related to the potential barrier, with low flux at low gate currents and high flux at higher currents, enabling conductance modulation in MoO_x.

When regulating device conductance, increasing the bias time continuously at a high I_g can result in the formation of hydrogen gas (**Figure S3b**). Electrocatalytic devices accelerate reactions like HER by applying potentials to drive reactant adsorption and charge transfer. Key metrics include Tafel slope (kinetics), overpotential (efficiency), and current density (activity).

To clarified the reversibility between the electrocatalytic and ECRAM regimes, we conducted conductance program tests on ECRAM devices that have undergone hydrogen evolution reactions (**Figure S4a**). After operating in the electrocatalytic regime at high proton flux (Q_H=2.7×10^{17} cm^{-2}), where bubble formation confirms hydrogen evolution (**Figure S4b,c**), we reduced the proton flux and performed ECRAM conductance regulation using gate pulses (±2 μA, 100 ms). The conductance of the MoO_x film was effectively modulated from 10 nS to 760 nS (**Figure S4d**), demonstrating the device's ability to transition back to the ECRAM regime. This reversible modulation between high conductance in ECRAM and efficient catalytic performance highlights the stability and functional versatility of MoO_x.

ECRAMs based on $MoO_{2.96}$ or $MoO_{2.64}$ were stimulated with I_g pulses of varying amplitudes, widths, and numbers. The data are fitted as shown in **Figure S5a-f**. Protons from Nafion are attracted towards the Nafion/MoO_x interface, where they react with MoO_x. When the gate current bias I_g is positive, this results in potentiation in conductance; the reverse process occurs for negative I_g, resulting in depression in conductance. Under the same pulse condition, the $MoO_{2.64}$ ECRAM shows a lower G_{max}/G_{min} ratio compared with the $MoO_{2.96}$ ECRAM. The fitting model is first calculated the interfacial cation concentration as $c_i = (c_{i0} - c_{i\infty})\exp\left[-(t/\tau_1)^{\alpha}\right] + c_{i\infty}$, where c_{i0}, $c_{i\infty}$, τ_1 and α are the initial cation concentration, the quasi-steady state cation concentration, time constant and exponential factor for the migration process, respectively.[1] The $c_{i\infty}$, τ_1 are described as $c_{i\infty} = c_{i,max}\left[1 - \beta\exp\left(-I_g/I_0\right)\right]$ and $\tau_1 = \gamma/I_g$, where β, I_0, and γ are constants, $c_{i,max}$ is the maximum cation concentration and I_g is the gate injection current. When the c_i exceeds a threshold value c_{th}, the excess ions are injected and chemically react with the channel. Then, the electron concentration is calculated as $\Delta c_e = \Delta c(H_nMoO_3) = c_p = (c_i - c_{th})\left(1 - e^{-(t-t_{th})/\tau_2}\right)$, where c_p, t_{th} and τ_2 are cation concentration, the threshold time for c_i to exceed c_{th} and the time constant for the Volmer step reaction, respectively.[2] The t_{th} can be calculated by $t_{th} = -\tau_1 \ln[(c_{th} - c_{i\infty})/(c_{i0} - c_{i\infty})]$. Finally, the channel conductance is calculated as $G(t) = \begin{cases} G_0 + e\mu_n \Delta c_e Wd/L, & t > t_{th} \\ G_0, & t \le t_{th} \end{cases}$, where e, μ_n, L, W, d, c_e, and G_0 are the electron charge, the electron mobility, channel length, the channel width, channel thickness, electron concentration, and the initial conductance ($G_0 = e\mu_n c_{e0} Wd/L$), respectively. In the range below saturation and above the threshold of cation concentration, the conductance can be described by the power law. Under pulse stimulation, G is potentiated by positive pulses $G_{potent} = G(t = mt_w)$ and de-potentiated by

negative pulses $G_{depre} = G(t = m_{max}t_w - m't_w)$, where m_{max} and m' represent the maximum numbers of positive and negative pulses, respectively, and t_w is the pulse width. The endurance test for the $MoO_{2.96}$ ECRAM (**Figure S5g**) showed that during 10 repeated cycles, the ratio of G_{max}/G_{min} remained high, above 100. For the sampled-current-voltammetry, the currents of devices with a metal-MoO_x-Nafion-metal structure were measured at various potential biases (**Figure S8**). The *I*-*t* currents biased at 1.4-2.0 V for the $MoO_{2.64}$ show close values, indicating that the current is saturated due to diffusion limitations.

As comparison, **Table S3** summarizes the performances of devices based on phase-transition material. **Table S4** shows the different types of devices based on MoO_x, suggesting that MoO_x is a key material in electronics and energy devices, widely used in optoelectronics (e.g., OLEDs, QLEDs, perovskite solar cells), energy storage (supercapacitors, Li-ion batteries), and catalysis (HER, CO_2 reduction). Its phase transitions are essential to unify these applications and mitigate performance degradation. MoO_x exhibits sensitive proton responsiveness, enabling dynamic conductance modulation without defect engineering, and reversible phase transitions, preserving device longevity. Molybdenum oxides (MoO_x) and MoO_x enriched with oxygen vacancies (O_v) were characterized using X-ray photoelectron spectroscopy (XPS). The valence states of molybdenum and oxygen elements were analyzed as shown in **Table S1**. The stoichiometric ratio of MoO_x was determined based on the molybdenum valence state distribution. MoO_x is referred to as $MoO_{2.96}$ for the regular form, and as $MoO_{2.64}$ for the O_v-rich form.

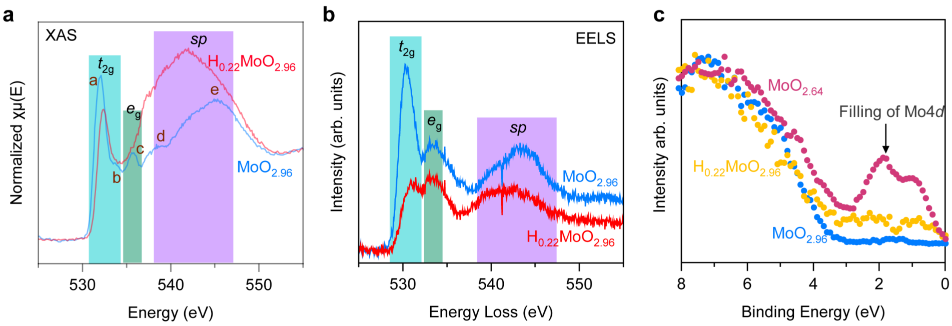

Figure S1. XAS O K-edge analysis and XPS analysis. (a), Synchrotron radiation X-ray absorption spectra of the oxygen K-edge of $MoO_{2.96}$ (blue) and $H_{0.22}MoO_{2.96}$ (red). (b), Energy-loss near-edge structure spectra of the O K-edge of $MoO_{2.96}$ (blue) and $H_{0.22}MoO_{2.64}$ (red). (c)**.** Valence band spectrum by XPS. The intensity of photoelectron emission in the valence band of $MoO_{2.96}$ (blue), $MoO_{2.64}$ (red) and $H_{0.22}MoO_{2.96}$ (yellow), measured by X-ray photoelectron spectroscopy.

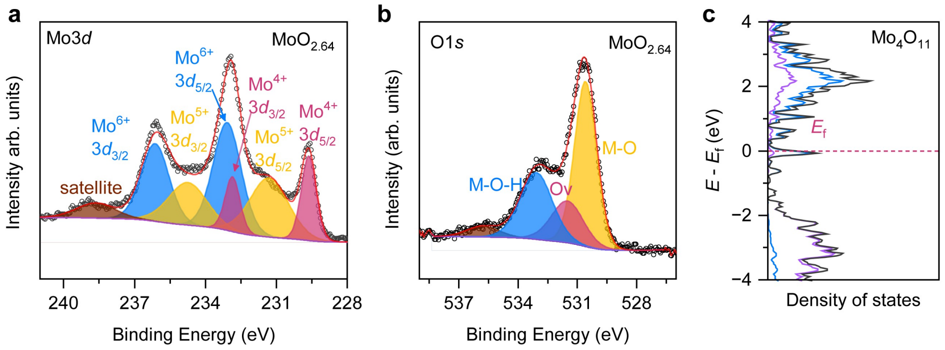

Figure S2. The property of the oxygen vacancy (O_v) rich molybdenum oxide. XPS core-level spectra of

Mo3*d* (a) and O1*s* (b) for $MoO_{2.64}$. (c), Density of states for Mo_4O_{11}.

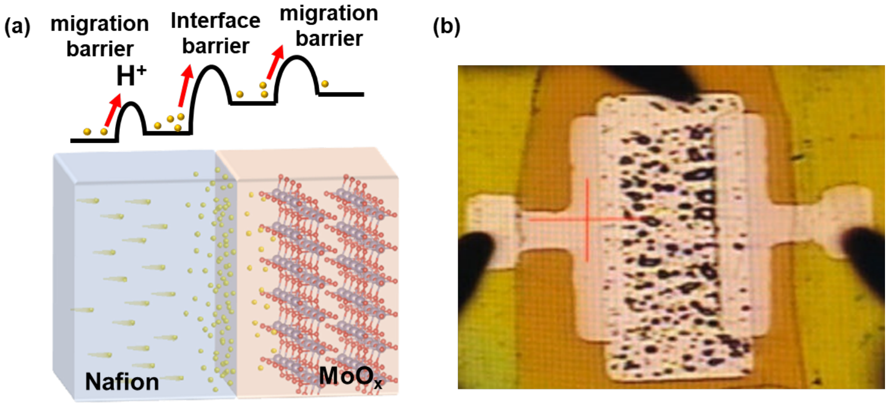

Figure S3. The proton migration and hydrogen evolution process. (a) The proton migration barrier and interface barrier in the Nafion/MoO_x. (b) The hydrogen gas generation in the ECRAM device at about 10^{17} cm^{-2} of Q_H.

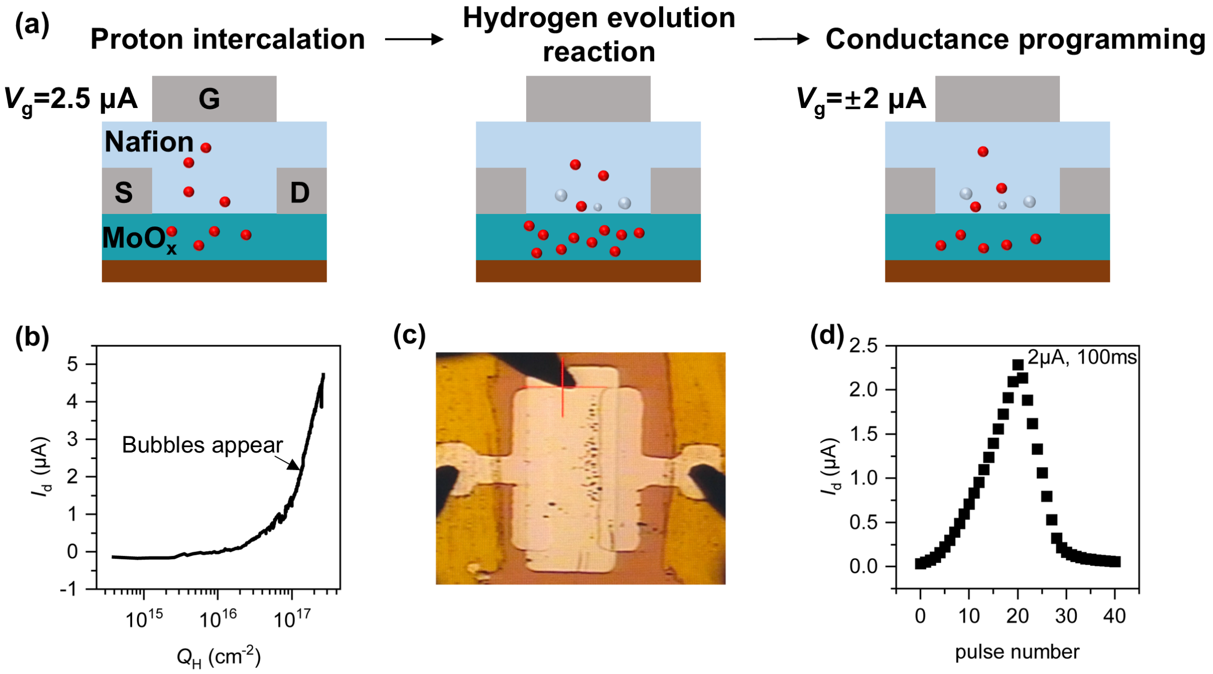

Figure S4. The reversible modulation between electrocatalytic and ECRAM regimes. (a) Test sequence of the device. First, a constant gate current was applied for inject the proton. Second, once bubbles are generated, the gate current was stopped. Third, after adjusting the conductance to a smaller value, gate current pulses were applied to increase and decrease the conductance. (b) The I_d-Q_H curve measured at I_g=2.5 μA, V_d=3 V, corresponding to the first step in (a). (c) the microscope photo of the device, corresponding to the second step in (a). (d) The reversible modulation of MoO_x channel conductance with the continuous gate pulses (±2 μA, 100ms), corresponding to the third step in (a).

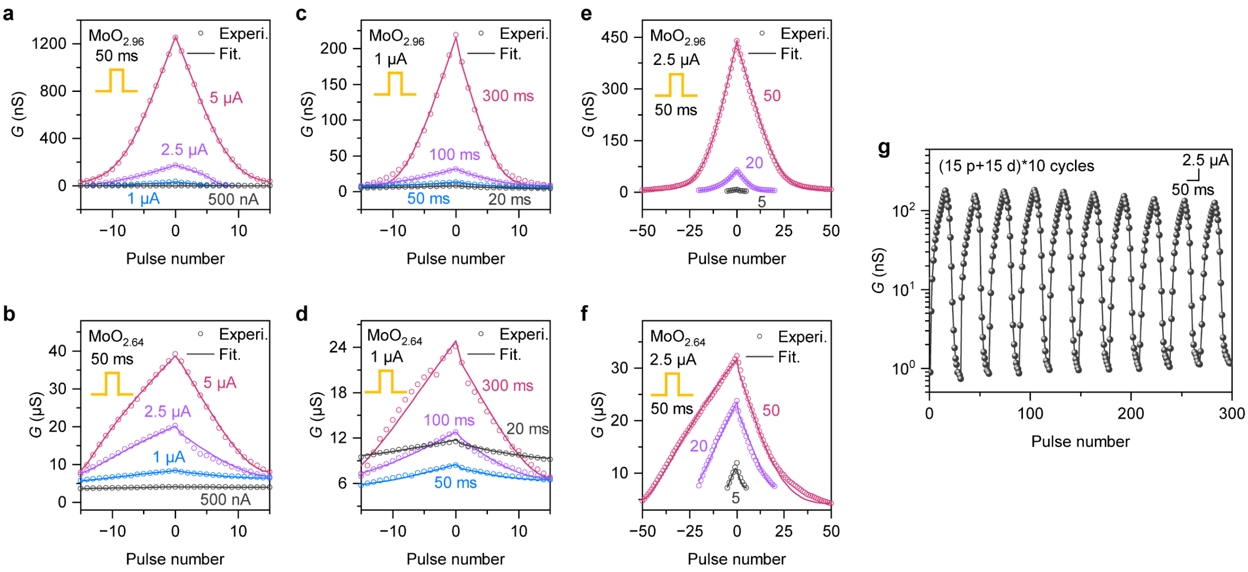

Figure S5. Device model fitting and endurance test. Fitting results of conductance modulation by bipolar pulses stimulation for $MoO_{2.96}$ (a, c, e, g) and $MoO_{2.64}$ (b, d, f) in different amplitudes (a, d), current pulse widths (c,d), pulse numbers (e, f). The hollow dot is the experimental (experi.) data, and the solid line is the fitting curve. (g) Endurance test showing a switching ratio of 100 over 10 cycles, each with 15 potentiation and 15 depression pulses (±2.5 μA, 50 ms).

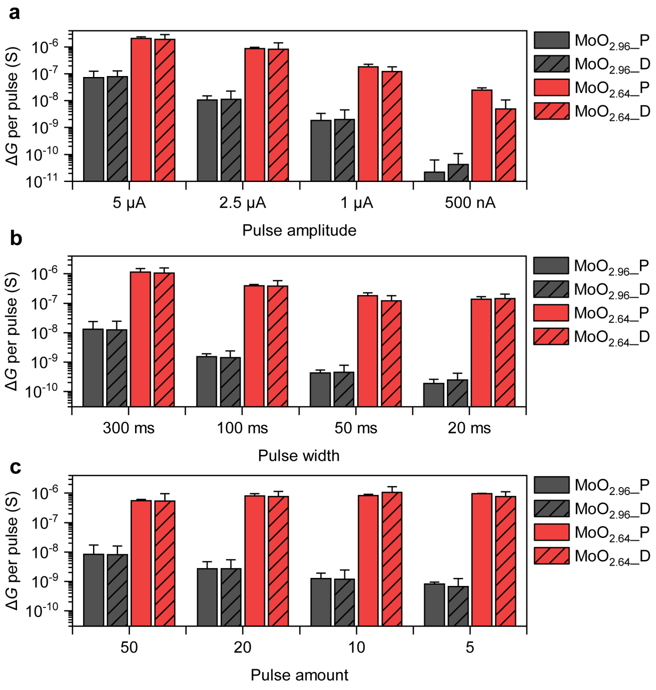

Figure S6. Corresponding average Δ*G* per pulse stimulation step for $MoO_{2.96}$ and $MoO_{2.64}$ channel with various pulse condition. (a) various pulse amplitudes. (b) various pulse widths. (c) various pulse amounts. The label P means the potentiation process and label D means the depression process. The error bars denote the data extracted from multiple pulse measurements (5 or more times) and presented as mean values ± standard deviation.

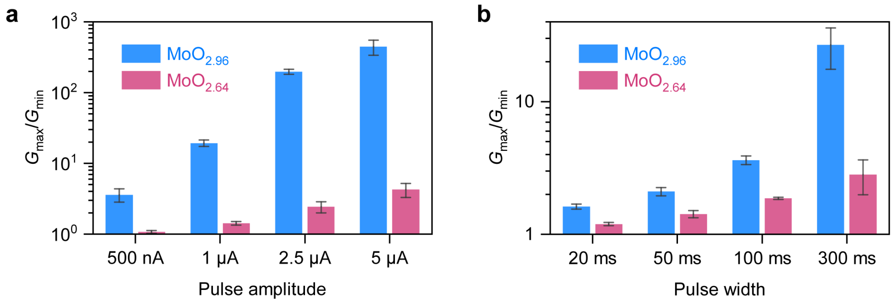

Figure S7. The G_{max}/G_{min} of the devices with different amplitudes (a), widths (b). The switching ratio of different pulse amounts are shown in the Fig. 3f in the main text. The representative data can refer to Fig. S5. The error bars denote the data extracted from three measurements and presented as mean values ± standard deviation.

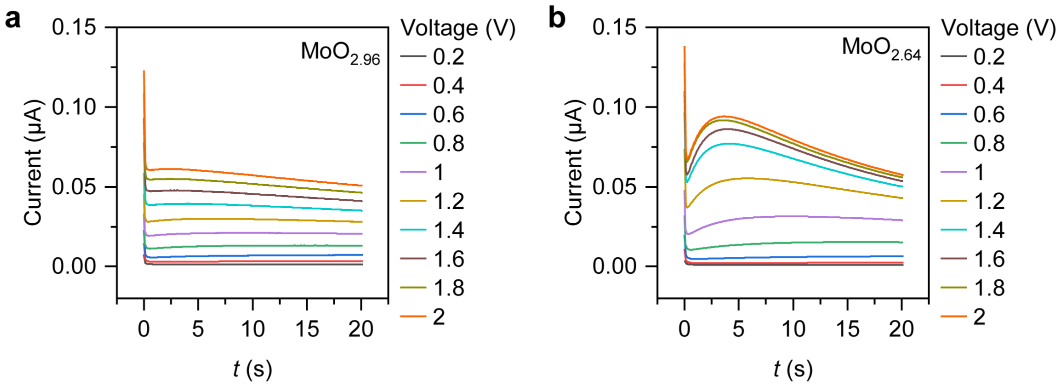

Figure S8. The *I-t* curves for the metal-MoO_x-Nafion-metal device at various voltage. (a) $MoO_{2.96}$ (b) $MoO_{2.64}$. The currents at 20 s are used plot the *I-V* scatter in figure 3e in the main text.

Table S1. Mo 3*d* subpeaks position and atomic percentages in XPS

	Mo^{6+} 3$d_{5/2}$	Mo^{5+} 3$d_{5/2}$	Mo^{4+} 3$d_{5/2}$	Mo^{6+}: Mo^{5+}: Mo^{4+}	Mo-O: O_V: M-O-H
$MoO_{2.96}$	233.21 eV	231.93 eV	/	92:8:0	68: 12: 20
$MoO_{2.64}$	233.08 eV	231.31 eV	229.63 eV	46:35:19	51: 19: 30
$H_{0.22}MoO_{2.96}$	232.75 eV	231.29 eV	/	70:30:0	29: 27: 44

Table S2. The calculated lattice constant b from the XRD

		2θ	d (Å)	calculated b (Å)	Δb (Å)
(020)	$MoO_{2.96}$	12.74°	6.943	13.885	0.132
	$H_{0.22}MoO_{2.96}$	12.62°	7.009	14.017	
(040)	$MoO_{2.96}$	25.67°	3.468	13.870	0.194
	$H_{0.22}MoO_{2.96}$	25.31°	3.516	14.064	
(060)	$MoO_{2.96}$	38.96°	2.310	13.859	0.195
	$H_{0.22}MoO_{2.96}$	38.40°	2.342	14.054	

Table S3. Summary of performances of devices based on phase-transition material.

	Method	G_{on}/G_{off} & testing environment	Retention time	Switch time	Application
MoO_x /Nafion (ECRAM) (this work)	atomic layer deposition	10^5	10^3 s	10^{-5} s	Rank-order-coding SNN
α-MoO_3/ionic liquid (synaptic transistor)[3]	mechanical exfoliation	3.4 @ 45%RH	50 s	10^{-3} s	N/A
α-MoO_3/ $LiClO_4$/PEO (synaptic transistor)[4]	mechanical exfoliation	17 @ 10^{-5} Torr	1.5×10^2 s	10^{-3} s	Simulated ANN
$MoS_{2-x}O_x$ (memristors)[5]	mechanical exfoliation	10^2	10^5 s	10^{-7} s	N/A
MoS_2/PVA (electrolyte gated transistor)[6]	mechanical exfoliation	~10^4 @ 50%RH	~1 s	10^{-2} s	"OR", "AND" logic
MoS_2/n-butyl Li solution (synaptic transistor)[7]	mechanical exfoliation	10^3 @ vacuum	7×10^3 s	10^{-3} s	N/A
MoS_2/Na^+-SiO_2 (synaptic transistor)[8]	mechanical exfoliation	10^6 @ 7×10^{-3} mbar	2.5×10^2 s	10^{-1} s	Simulated ANN
WO_3/ionic liquid (electrolyte gated transistor)[9]	pulsed laser deposition	~10^4	40 s	7×10^{-2} s	N/A
WO_3/LiPON (ECRAM)[10]	-	10^3	~10^3 s	5×10^{-9} s	N/A
W/WO_{3-x} (synaptic device)[11]	sputter	10	~10 s	10^{-2} s	Simulated ANN
WO_3/HfO_2 (ECRAM)[12]	-	~20	5×10^4 s	10^{-8} s	Simulated ANN
$WO_{2.7}$/Li_3PO_4 (electrolyte gated	sputter	~7	10^2 s	0.5 s	N/A

transistor)[13]					
WO_3/Nafion (electrolyte gated transistor, 100% relative humidity)[14]	sputter	10^7 @ 100%RH	~2×10^2 s	5×10^{-3} s	N/A
VO_2/ionic liquid (electrolyte gated transistor)[15]	pulsed laser deposition	~2×10^2	~3×10^3 s	2×10^{-1} s	Simulated ANN
Nb_2O_5/Li_xSiO_2 (electrolyte gated transistor)[16]	sputter	3×10^2	10^3 s	10^{-7} s	STDP-based SNN

Table S4. The devices composed of molybdenum oxide material.

Device type	Material
OLED[17]	ITO/**MoO_3**/Ir(mppy)$_3$ TCTA/TPBi/LiF/Al
QLED[18]	ITO/**MoO_3**/Poly-TPD/QDs/TPBi/LiF/Al
PeLED[19]	ITO/**MoO_3**/$PEA_2(FAPbBr_3)_2PbBr_4$/TPBi/LiF/Al
Perovskite Solar cell[20]	Ag/BCP/C60/PM6Y6: PCBM/**MoO_x**/ITO
dye-sensitized solar cells[21]	FTO/TiO_2/N719/**MoO_3**/MoS_2
Organic solar cell[22]	ITO/**MoO_3**/P3HT: PCBM/TiO_2/ITO
Organic photovoltaic devices[23]	P3HT: PCBM/**MoO_3**
Photodetector[24]	$CsPbBr_3$/**MoO_3**
P-type OFET[25]	Al/ **MoO_3**/CuPc/SiO_2/p++-Si
Surface Phonon Polaritons [26]	**MoO_3**/SiC
Heterojunction catalysis[27]	MoS_2@**MoO_3**

Supplementary Note 2: DFT calculation.

First of all, a standard MoO_3 crystal structure composed of 4 Mo atoms and 12 O atoms was investigated by Vienna Ab initio Simulation Package (VASP). The relaxed lattice parameters are in reasonable line with the experimental value of 3.963 Å, 13.856 Å, and 3.697 Å[28]. The calculated *b* parameter, which is slightly larger than the experimental result, can be attributed to the limitation that the exchange–correlation function underestimates the Van der Waals (VdW) interaction forces[29]. In this calculation, the long-range VdW interaction is described by the DFT-D3 approach[30] and the calculated *b* parameter is relatively close to the experimental data than the previous report[31]. Three different sites of oxygen vacancy displayed in **Figure S9** were considered, namely, the O_t, O_a or O_s site. The formation energy E_{for} is computed by:

$$E_{\mathrm{for}} = E_{\mathrm{Mo4O11}} - (E_{\mathrm{Mo4O12}} - E_{\mathrm{O}})$$

where E_{Mo4O11}, E_{Mo4O12} and E_{O} are the total energies of Mo_4O_{11}, Mo_4O_{12} and one O atom, respectively. The $O_{t\text{-vacancy}}$ configuration exhibits the lowest formation energy of 0.284 eV/atom (**Table S6**).

Furthermore, Mo_4O_{12} and O_v-rich Mo_4O_{11} with one proton intercalation were studied. Ten different adsorption sites and orientations were considered in HMo_4O_{12}, as demonstrated in **Figure S10**. The $O_{t\text{-}t2}$ configuration exhibits the lowest formation energy of -2.751 eV/atom (**Table S7**). For HMo_4O_{11}, as demonstrated in **Figure S11**, eleven different adsorption sites and orientations were considered. The $O_{t\text{-}s}$ configuration exhibits the lowest formation energy of -2.485 eV/atom (**Table S8**), indicating that the $O_{t\text{-}s}$ configuration is the most stable site, which is consistent with previous calculation results[32]. The energy band structure and density of states for MoO_3, Mo_4O_{11}, HMo_4O_{12}, and HMo_4O_{11} were calculated, as demonstrated in **Figures S12** and **S14**. The t_{2g} and e_g orbitals split from the Mo4d, and p_x, p_y, and p_z orbitals split from O2p and O1s were calculated, as demonstrated in **Figure S15**. Bader charge analysis was utilized to assess the atomic charges and charge transfer in heterostructures, as shown in **Figure S16**. The hydrogen adsorption energy at different H/Mo ratios was calculated (**Figure S17**).

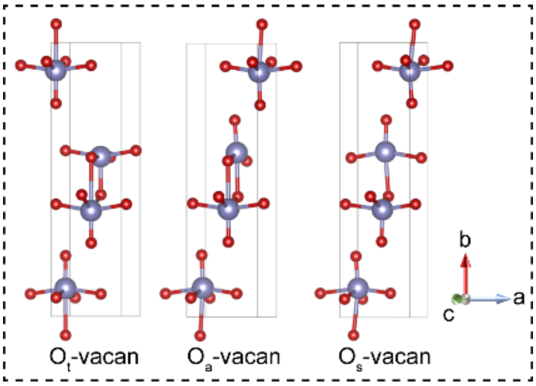

Figure S9. Schematic of the oxygen vacancy site.

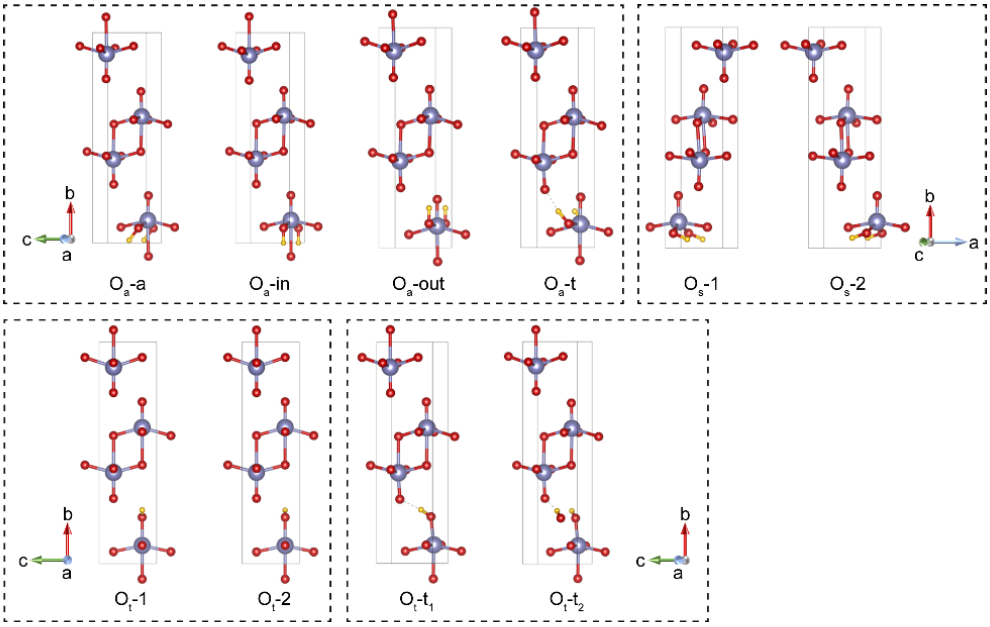

Figure S10. Schematic of the hydrogen adsorption site in Mo_4O_{12}.

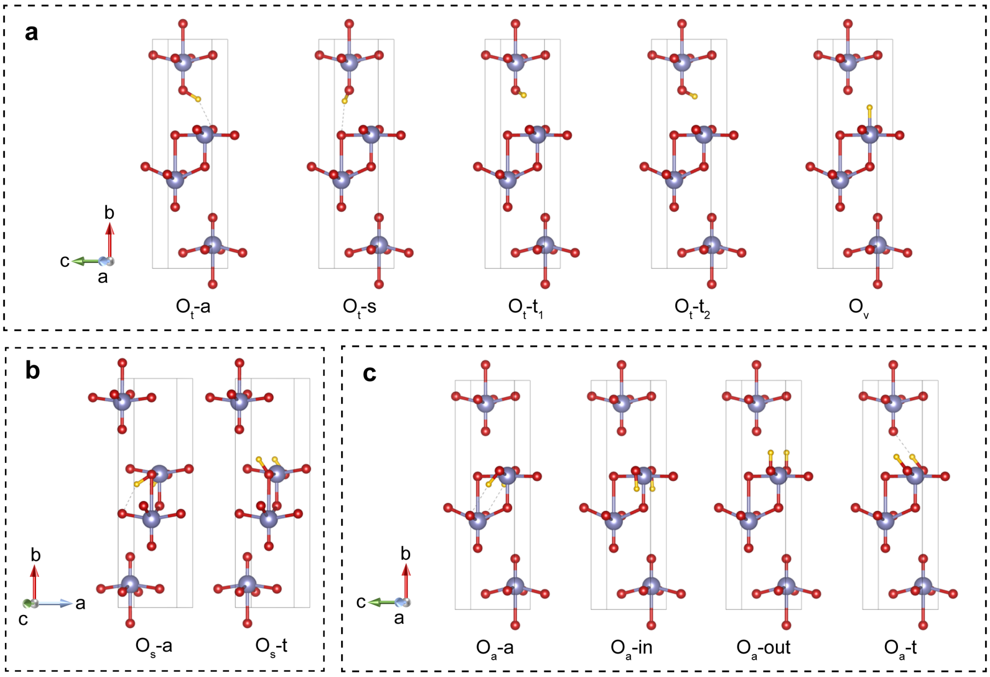

Figure S11. Schematic of the hydrogen adsorption site in O_v-rich Mo_4O_{11}. (a) terminal sites O_t, (b) symmetric sites O_s, (c) asymmetric sites O_a.

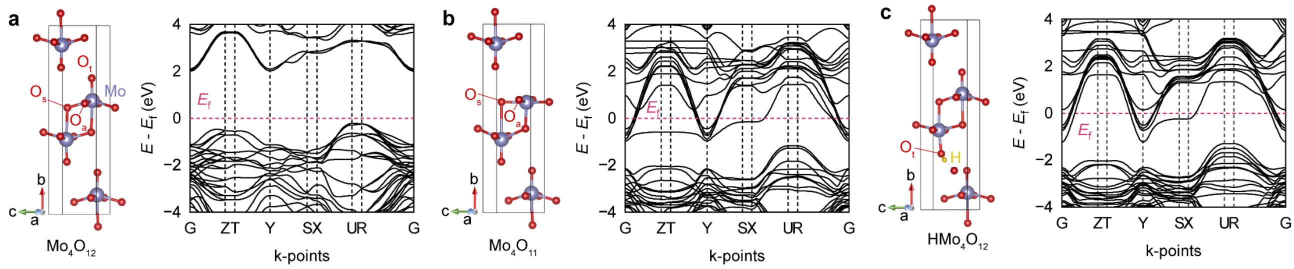

Figure S12. DFT calculated steady crystal structures and band structure for MoO_3 (a), O_v-rich Mo_4O_{11} (b) and HMo_4O_{12} (c). The α-MoO_3's orthorhombic structure comprises interconnected MoO_6 octahedra via Van der Waals (VdW) forces along the b-axis. It features three symmetrically equivalent oxygen sites (O_s), one terminal oxygen (O_t) facing the VdW gap, and two asymmetric oxygen (O_a) sites with varying Mo−O bond lengths. In HMo_4O_{12} (**c**), the most stable proton adsorption occurs at the $O_{t\text{-}t2}$ configuration (**Table S7**)[33], leading to an extension of the b parameter and electron filling of Mo4d states below the Fermi level (**Table S5**).

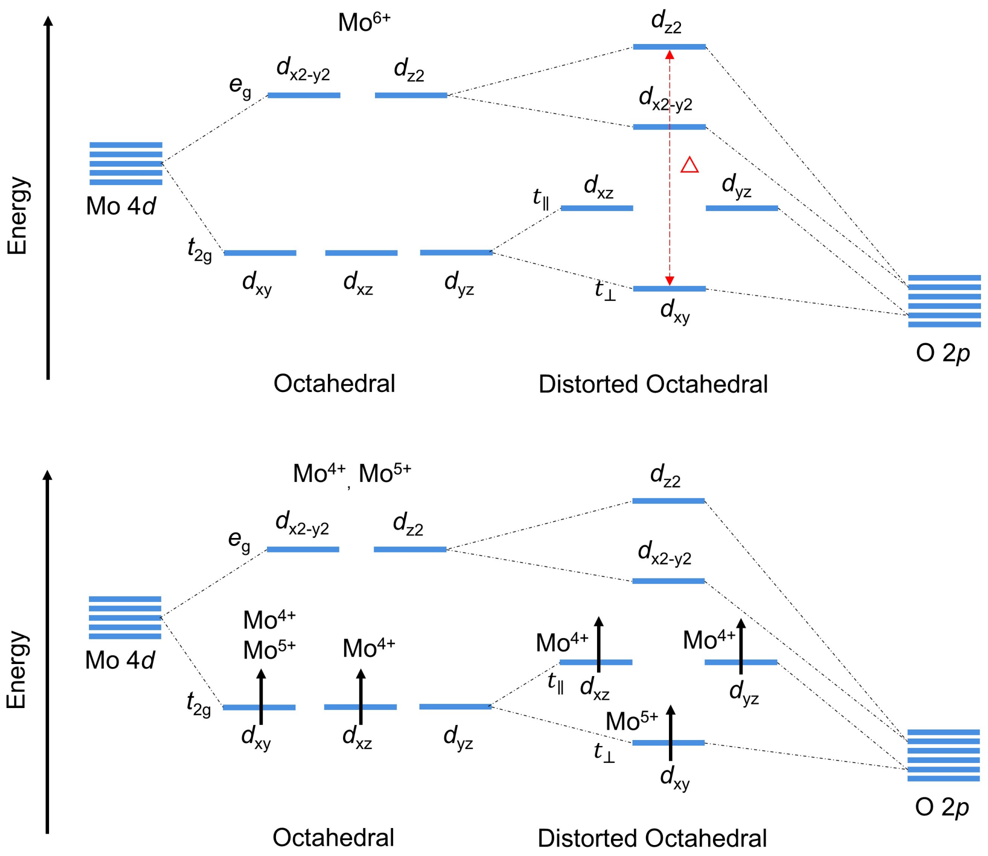

Figure S13. The Mo4d and O2p hybrid orbitals and the formation of the e_g, t_{2g} orbitals. For Mo^{4+} and Mo^{5+}, the electrons fill on the hybrid orbitals.

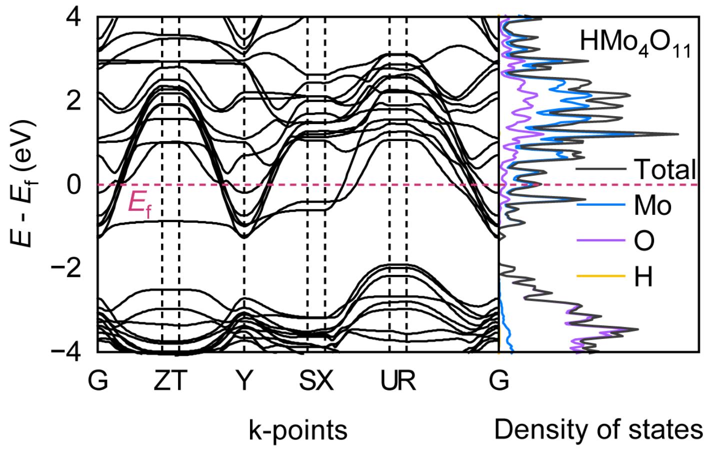

Figure S14. Energy band structure and density of states for HMo_4O_{11}.

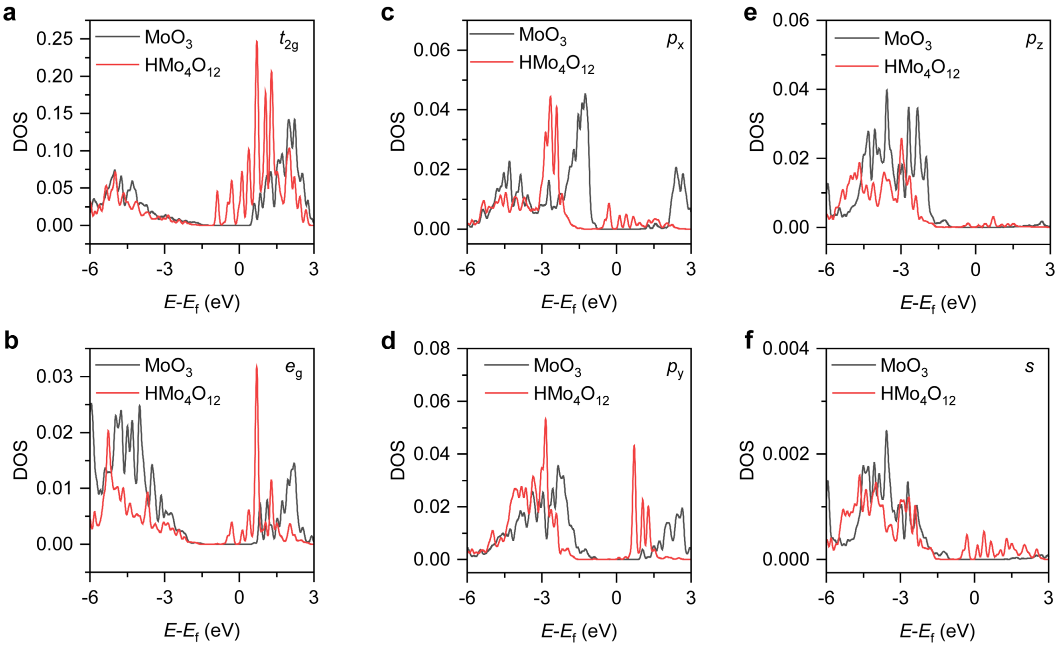

Figure S15. The partial density of states for MoO_3 and HMo_4O_{12}. The t_{2g} (a) and e_g (b) orbits split from the Mo 4*d*. The p_x (c), p_y (d), p_z (e) orbits split from O 2*p* and O 1*s* (f). The e_g state of Mo 4*d* bonds from -6 eV to -2 eV decreases after the proton intercalation.

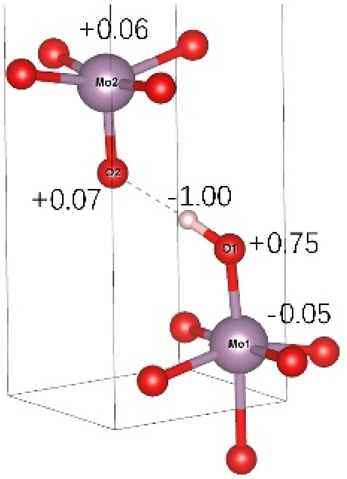

Figure S16. The Bader charge analysis between the MoO_3 and HMo_4O_{12}.

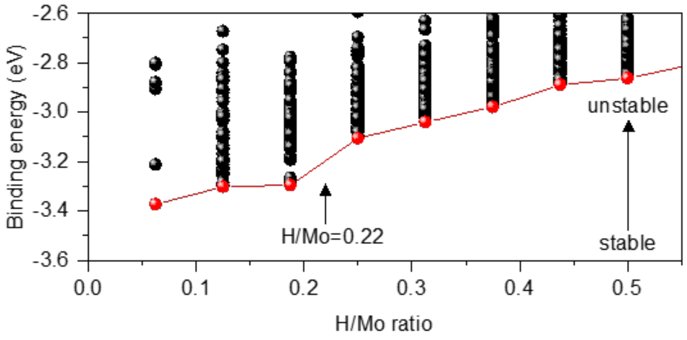

Figure S17. The hydrogen binding energy at different H/Mo ratios. The red dots indicate the structure with the lowest energy (more stable). The black dots represent the structures with other different adsorption sites. The ratio of H/Mo ranges from 0 to 0.5, approximately corresponding to Q_H ranging from 0 to 6.0×10^{16} cm^{-2}.

Table S5. The lattice constants calculated from density functional theory (DFT) compared with the powder diffraction file (PDF)

		L_a (Å)	L_b (Å)	L_c (Å)	Δb (Å)
DFT	Mo_4O_{12}	3.903	14.023	3.678	0.636
	HMo_4O_{12}	3.766	14.659	3.731	
PDF	MoO_3	3.962	13.858	3.697	0.208
	$H_{0.31}MoO_3$	3.881	14.066	3.728	

Table S6. Lattice parameters and formation energy of Mo_4O_{11} and Mo_4O_{12}

Mo_4O_{11}	a (Å)	b (Å)	c (Å)	α=β=γ	E_{for} (eV/atom)
$O_{t\text{-}vacan}$	3.730	14.056	3.784	90°	0.284
$O_{a\text{-}vacan}$	3.746	13.942	3.730	90°	0.376
$O_{s\text{-}vacan}$	3.789	14.364	3.626	90°	0.349
Mo_4O_{12}	3.903	14.023	3.678	90°	

Table S7. Lattice parameters and adsorption energy of HMo_4O_{12}

HMo_4O_{12}	a (Å)	b (Å)	c (Å)	α=β=γ	E_{ads}(eV/atom)
$O_{a\text{-}a}$	3.848	14.067	3.726	90°	-2.668
$O_{a\text{-}in}$	3.846	13.993	3.725	90°	-2.568
$O_{a\text{-}out}$	3.964	13.908	3.698	90°	-2.066
$O_{a\text{-}t}$	3.942	13.668	3.719	90°	-2.226
$O_{s\text{-}1}$	3.759	14.064	3.770	90°	-2.256
$O_{s\text{-}2}$	3.750	14.069	3.776	90°	-2.241
$O_{t\text{-}1}$	3.765	14.417	3.740	90°	-2.711
$O_{t\text{-}2}$	3.763	14.433	3.738	90°	-2.704
$O_{t\text{-}t1}$	3.767	14.271	3.739	90°	-2.735
$O_{t\text{-}t2}$	3.766	14.659	3.731	90°	-2.751
Mo_4O_{12}	3.903	14.023	3.678	90°	

Table S8. Lattice parameters and adsorption energy of HMo_4O_{11}

HMo_4O_{11}	a (Å)	b (Å)	c (Å)	α=β=γ	E_{ads} (eV/atom)
O_{a-a}	3.846	13.374	3.772	90°	-1.547
O_{a-in}	3.831	13.450	3.777	90°	-1.569
O_{a-out}	3.743	13.827	3.782	90°	-2.176
O_{a-t}	3.739	13.843	3.781	90°	-2.175
O_{s-a}	3.745	13.449	3.842	90°	-1.771
O_{s-t}	3.750	13.427	3.841	90°	-1.791
O_{t-a}	3.734	14.109	3.808	90°	-2.314
O_{t-s}	3.740	14.090	3.794	90°	-2.485
O_{t-t1}	3.741	13.990	3.792	90°	-2.479
O_{t-t2}	3.739	14.193	3.789	90°	-2.476
O_v	3.747	14.227	3.750	90°	-2.131
Mo_4O_{11}	3.730	14.056	3.784	90°	

Supplementary Note 3: The integration of the MoO_x ECRAM

For probing the response speed of the MoO_x ECRAM, gate potential pulses with various widths were applied. **Figure S18** shows the potential and current output from the semiconductor parameter analyzer when the width of the pulse is 10 μs. The detailed currents stimulated by the positive and negative pulses are shown in **Figure S19(a)-(e)**. The corresponding conductance is summarized in **Figure S19(f)**.

By integrating the drain electrode of the 1st ECRAM with the gate electrode of the 2nd ECRAM, the conductance (*G*) of the 1st ECRAM can be used to modulate the average change in *G* (Δ*G*) for the 2nd ECRAM stimulated by identical pulses. **Figure S20** shows the I_d-t curves of the 2nd ECRAM under continuous pulses (±1 μA, 10 ms) as input to the drain of the 1st ECRAM.

To characterize the light-writing and erasing memory, a commercial photodiode (PD) was used. The response time is about 30 μs, and the dark/illumination current ratio is about 2×10^5 (**Figure S21**). By comparison, the MoO_x ECRAM shows nearly no response under the same illumination (**Figure S22**). By connecting the PD with the gate electrode of the ECRAM in both forward and reverse directions, the light signal can be used to adjust *G* with response times of 60-80 μs (**Figure S23**). The experimental organic photodetector can replace the commercial PD to realize the light-writing and erasing functions (**Figure S24**).

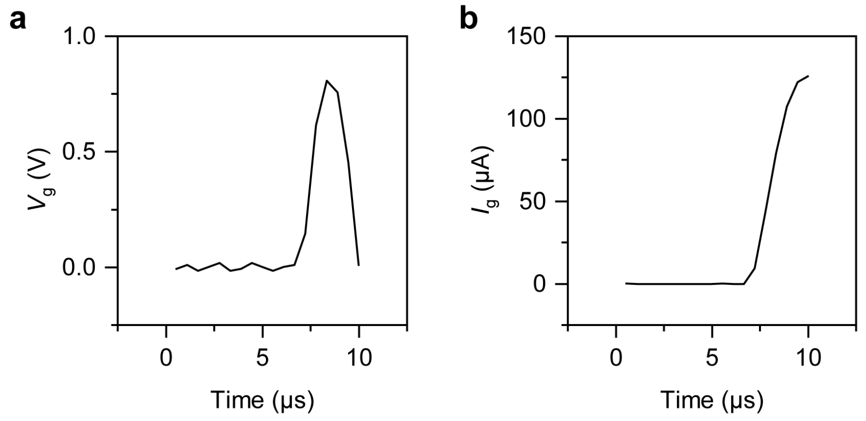

Figure S18. The short voltage pulse generated from the semiconductor parameter analyzer. The pulse amplitude is set as 10 V and width is 10 μs. (a) The V_g-t curve, (b) the I_g-t curve.

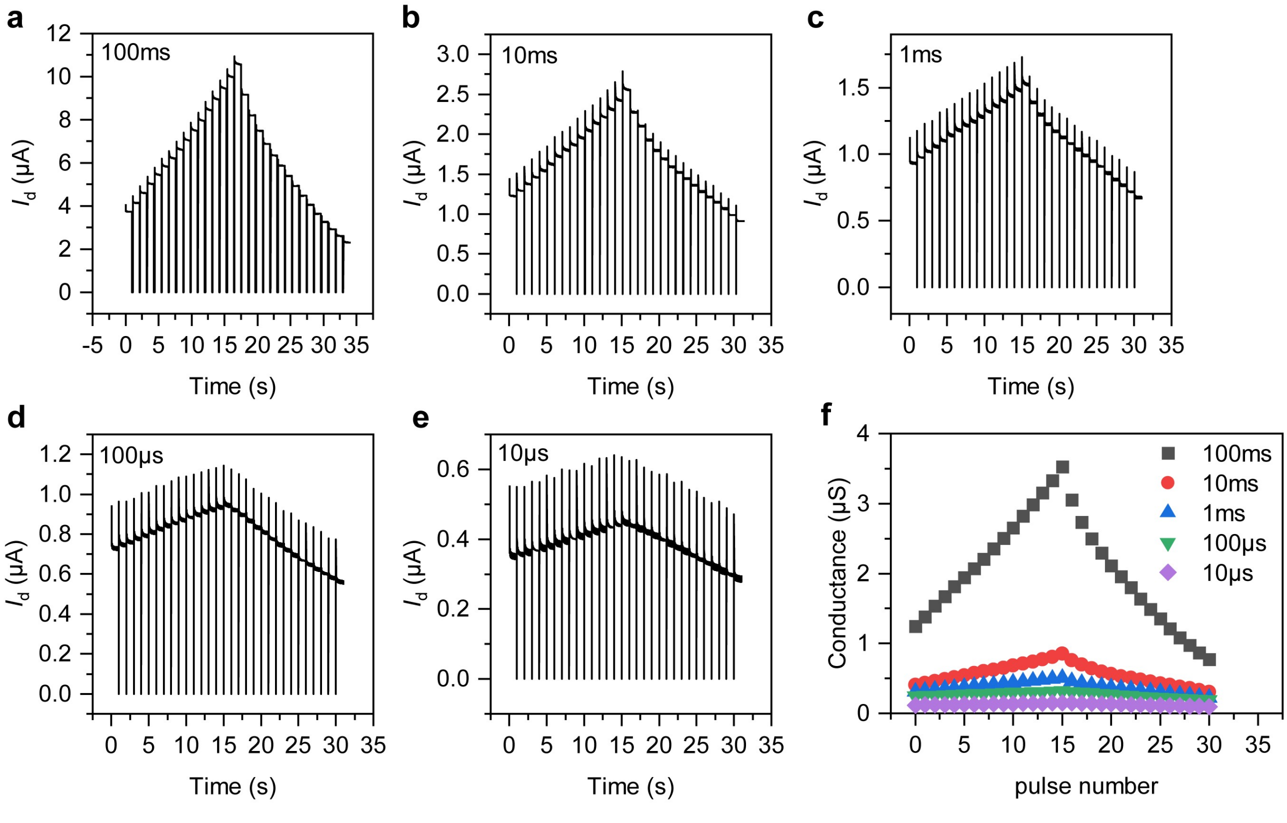

Figure S19. The programmed conductance by continuous gate pulses. The I_d-t curves at V_d=3 V programmed with constant gate pulse amplitude (±10 V) and various gate voltage pulse widths from 100 ms (a) to 10 μs (e). (f) The corresponding programmed conductance in (a)-(f).

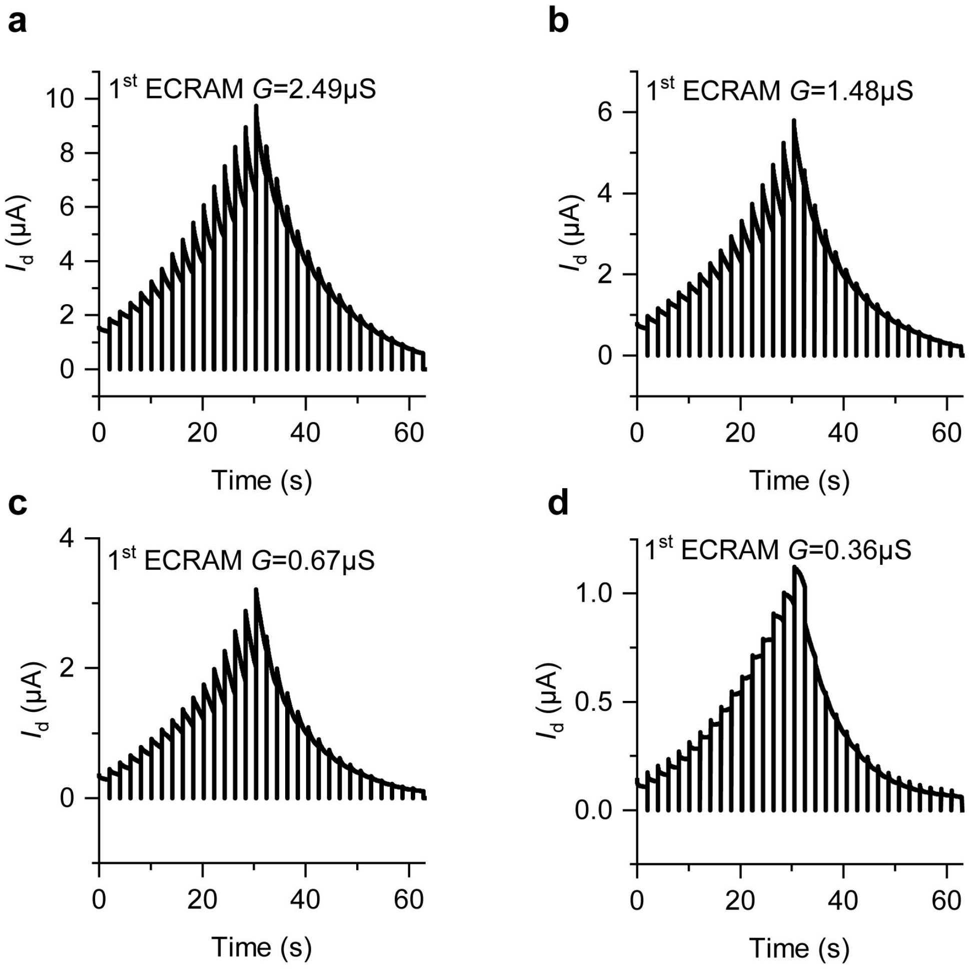

Figure S20. The programming conductance in the integration of two ECRAMs. The I_d-t curves of the 2nd ECRAM at V_d=5 V under continuous pulse (±1 μA, 10 ms) as input to the drain of the 1st ECRAM, whose conductance was adjusted from 2.49 μS (a) to 0.36 μS (d).

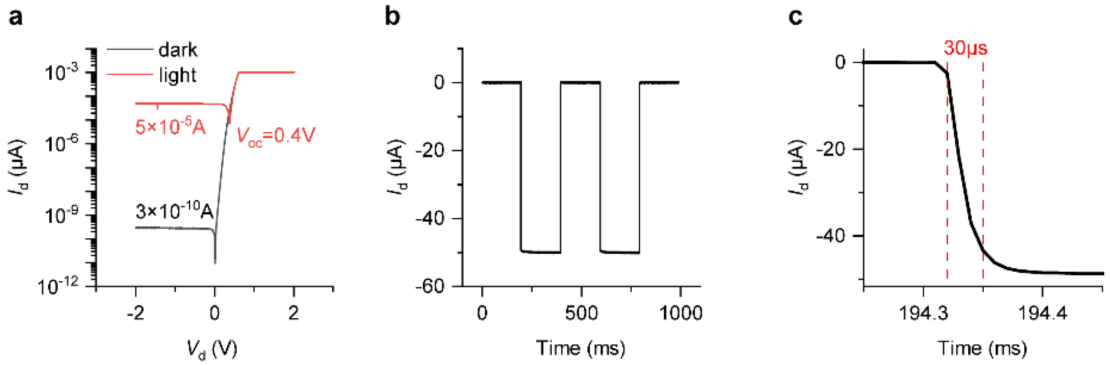

Figure S21. The commercial photodetector performance. (a) the I-V curves under the illumination (λ = 700 nm) and dark. (b) The I-t curve under the light pulse (λ = 700 nm, 0.2 s) and (c) the response time.

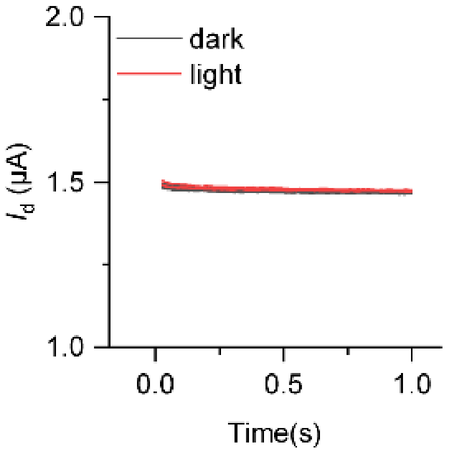

Figure S22. The light response of the MoO_x. The I_d-t curves of the MoO_x channel under the illumination (λ = 700 nm) and dark.

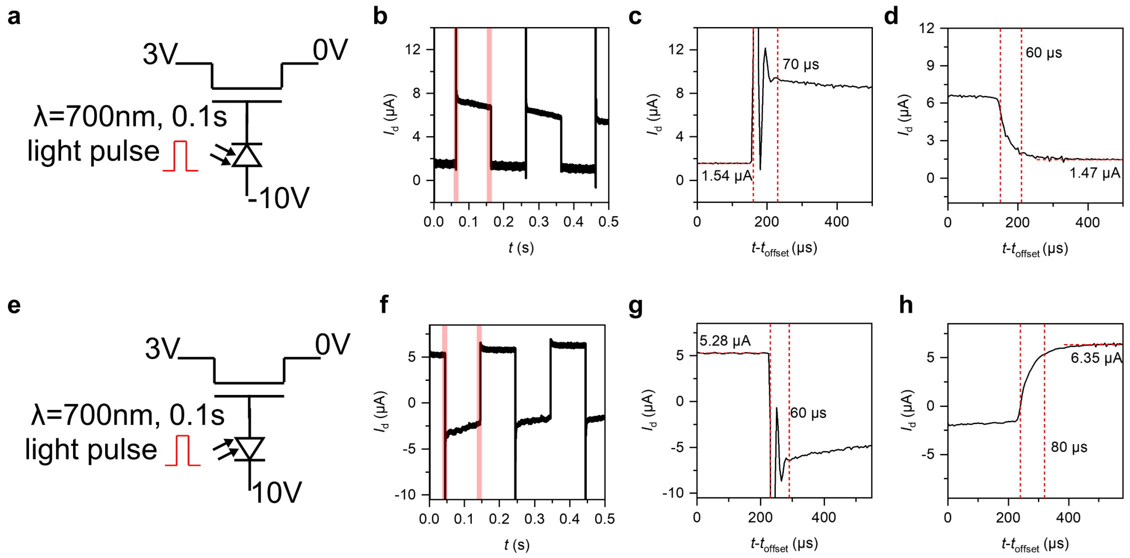

Figure S23. The fast light response of the ECRAM integrated with a photodetector (PD) at gate. (a)(e) Circuit schematic of ECRAM with photodetector at gate electrode connected with negative electrode (a) and positive electrode (e). The depression (b) and the potentiation (f) after the 0.1 s illumination (λ = 700 nm). (c-d), (g-h) The enlarged view of of I_d-t curves.

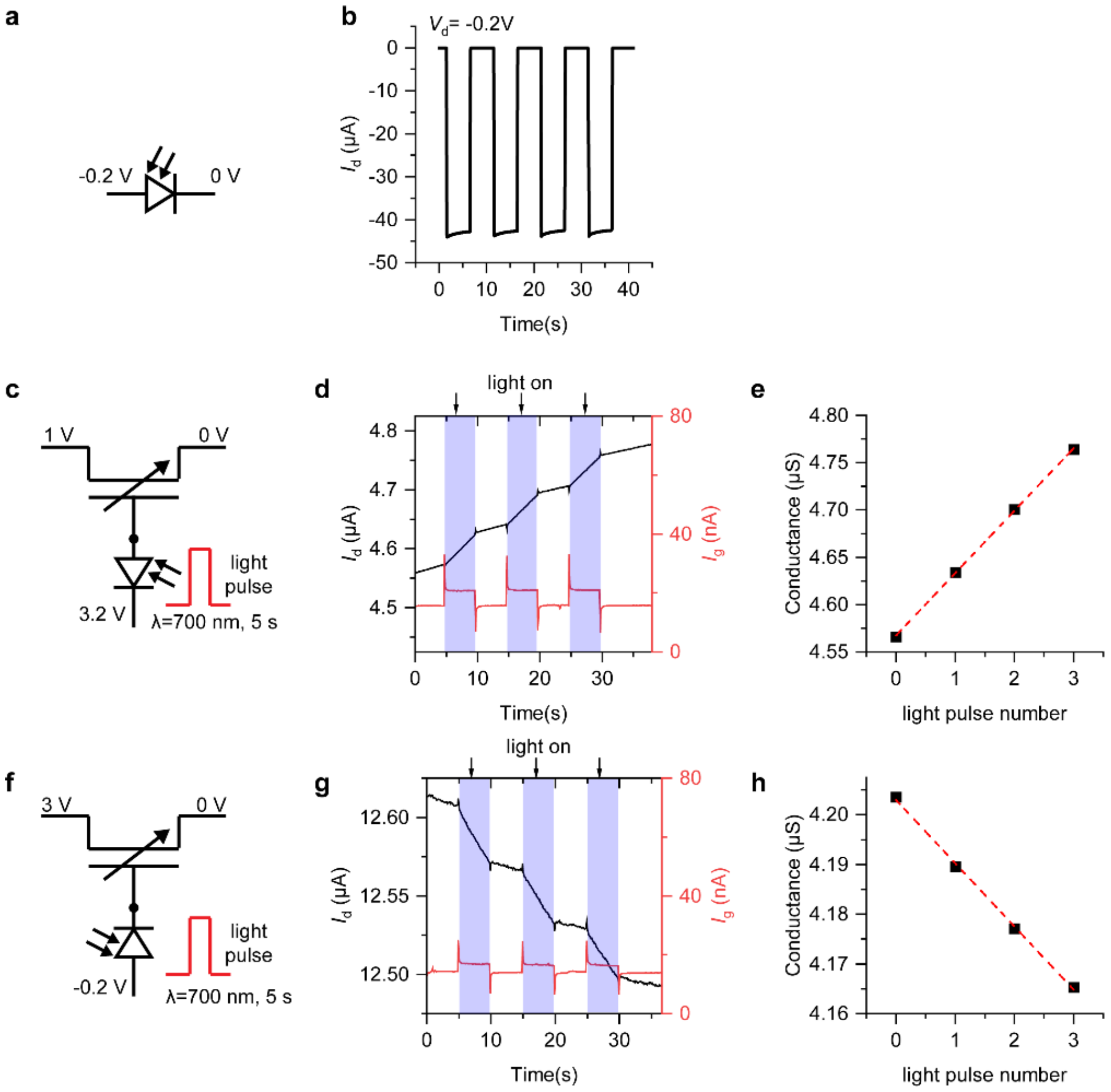

Figure S24. The performance of the organic photodetector and the integration with ECRAMs. (a) The bias of the device. (b) The I-t curve under the light pulse (λ = 700 nm, 5 s). (c)-(e) the circuit diagram, I_d-t curve, I_g-t curve and the conductance at potentiation case. (f)-(h) the circuit diagram, I_d-t curve, I_g-t curve and the conductance at depression case.

Supplementary Note 4: The hardware spiking neural network

The 30-device matrix panel was fabricated for the network as described in the main context. Among these 30 devices, 27 devices form 1st-stage synapses in the 9 × 3 structure, while the remaining 3 devices form the 2nd-stage neurons, accumulating the signals from 1st-stage devices. The 3×3 pixel images are introduced to two kinds of noise. The pixel-flipping noise inverts the white and black colors of each pixel, while grayscale noise results in increased or decreased grayscale values (**Figure S25**). These images are converted into pulses according to the temporal rank-order coding. The detailed flowchart and the symbol list are shown in **Figure S26** in the main text and **Table S9**, respectively. These signals are generated by the microcontroller and input to the device array (**Figure S27**). During training in software, **Figure S28** shows the evolution of the matrix conductance at various epochs, and **Figure S29** shows the accuracy and error trends. Based on the training results, the matrix conductance was updated to the target values. **Figure S30** shows the four conductance

states of 27 devices. The accurate setting of conductance states ensures the accuracy of neural networks. **Figure S31** shows the G/G_0-t response to pulses with different amplitudes and time delays. The change in G/G_0 increases with the total pulse amplitude, ensuring the accuracy needed to classify noisy images.

To compared the power consumption between the spiking neural network (SNN) proposed in this work and the artificial neural network (ANN), as shown in **Figure S32**, we evaluated the power consumption for performing one inference process. For the SNN, the power is calculated as $W = N_{1\mathrm{st}}(V_{\mathrm{p}}^2/R_{1\mathrm{st}})t_{\mathrm{pt}} + N_{2\mathrm{nd}}(V_{2\mathrm{nd}}^2/R_{2\mathrm{nd}})t_{\mathrm{total}}$, where $N_{1\mathrm{st}}$ is the device number (= pixel number × image type) for stimulating the pulse or potential inputs in the 1st-stage ECRAMs, V_{p} is the pulse amplitude, $R_{1\mathrm{st}}$ is the resistance for the device after programing, t_{pt} is the total pulse stimulation time, $N_{2\mathrm{nd}}$ is the device number (=image type) in the 2nd-stage ECRAMs, $V_{2\mathrm{nd}}$ is the pulse amplitude for the 2nd-stage ECRAMs, $R_{2\mathrm{nd}}$ is the resistance for the 2nd-stage ECRAMs, t_{total} is the total time used in one inference process. For the ANN, the power is calculated as $W = N_{1\mathrm{st}}(V_{\mathrm{bias}}^2/R_{1\mathrm{st}})t_{\mathrm{total}}$, where V_{bias} is the constant potential as input, the other parameters have the same definition in the SNN. The detail values are shown in **Table S10**. The error stick in **Fig. 4jk** in the main text was evaluated according to the variation of $R_{1\mathrm{st}}$.

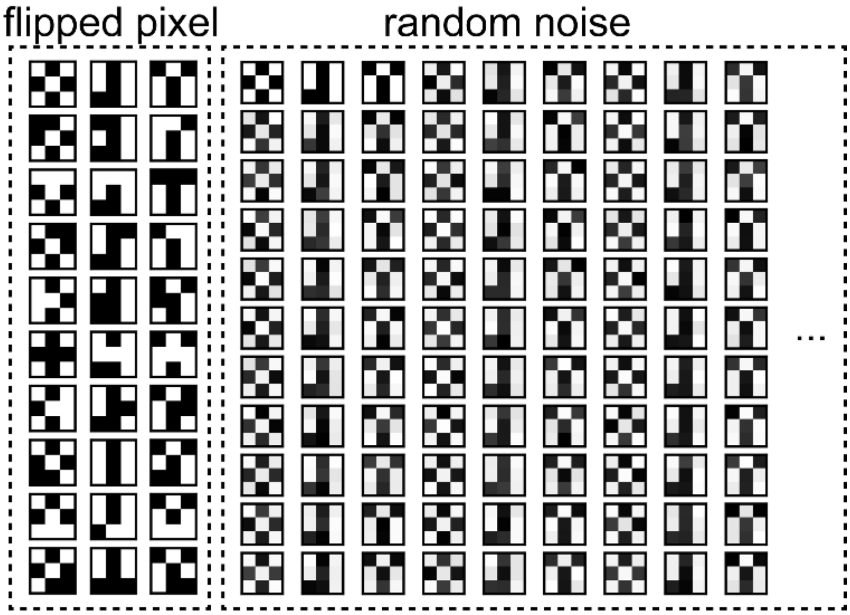

Figure S25. The image "o", "J", "Y" data sets for the spiking neural network. Left: with one flipped pixel. Right: with 20% random noise.

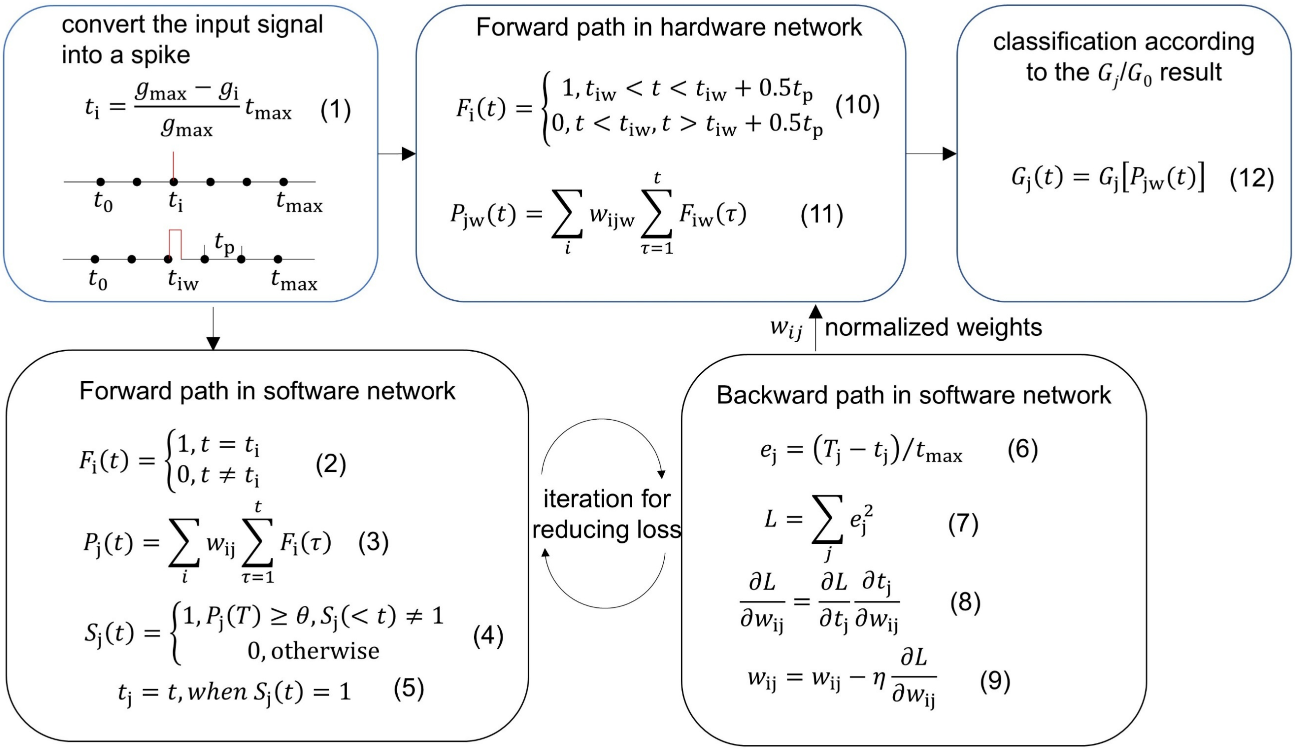

Figure S26. The flowchart of the implementation of the SNN.

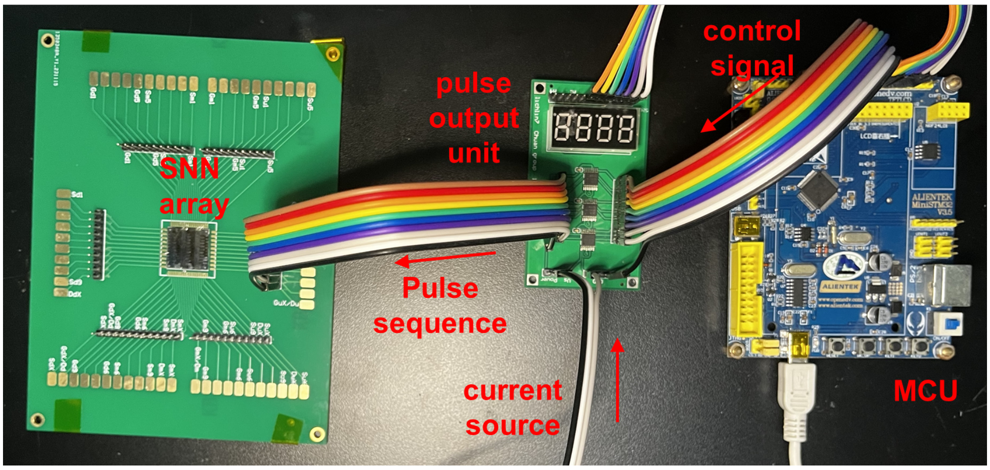

Figure S27. Photograph of the pulse sequence control setup.

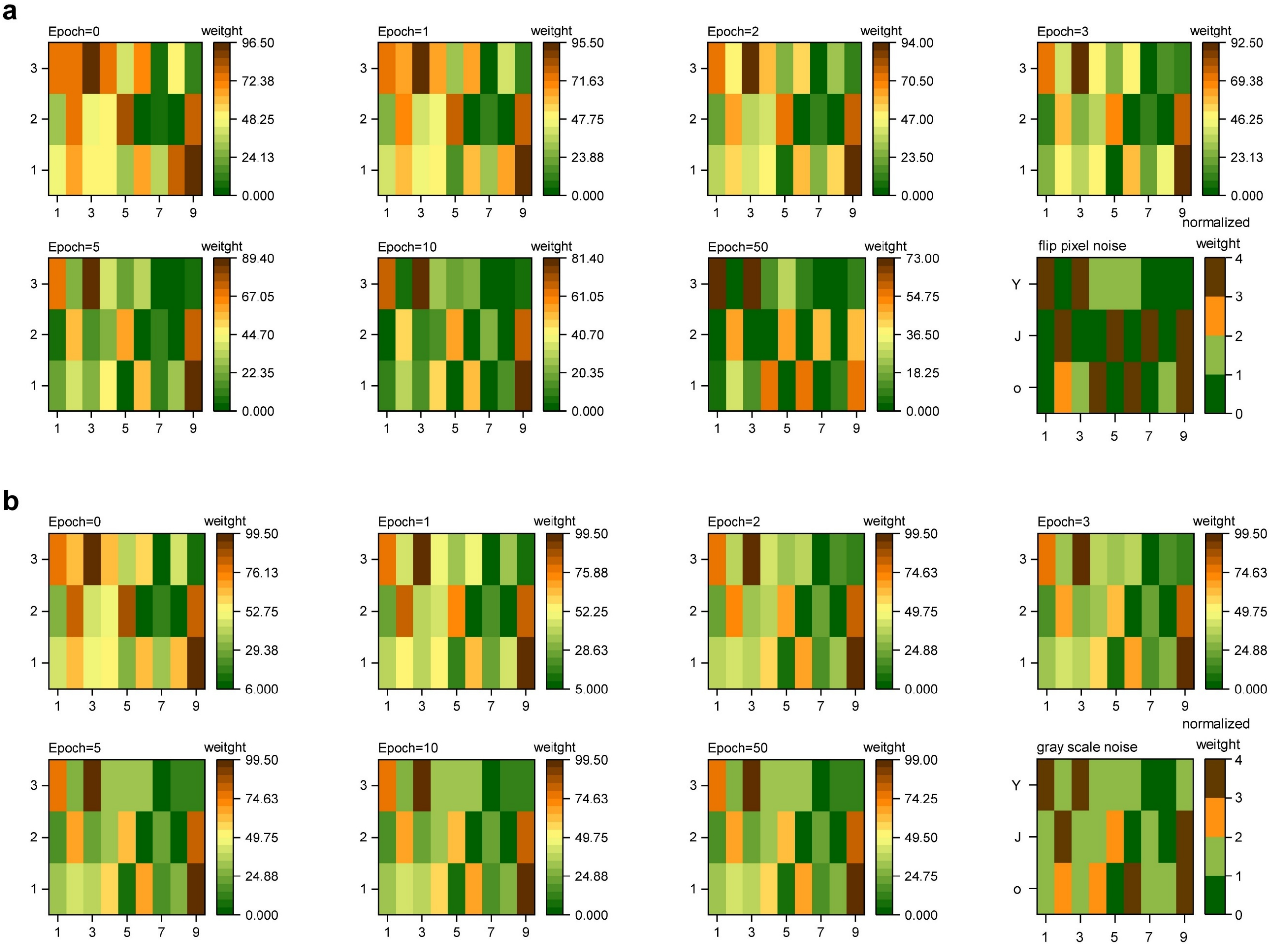

Figure S28. The weights of the spiking neural network evolution with training epoch. The weights from epoch 1 to epoch 50 and the final normalized weights for flipped pixel noise (a) and for random noise (b).

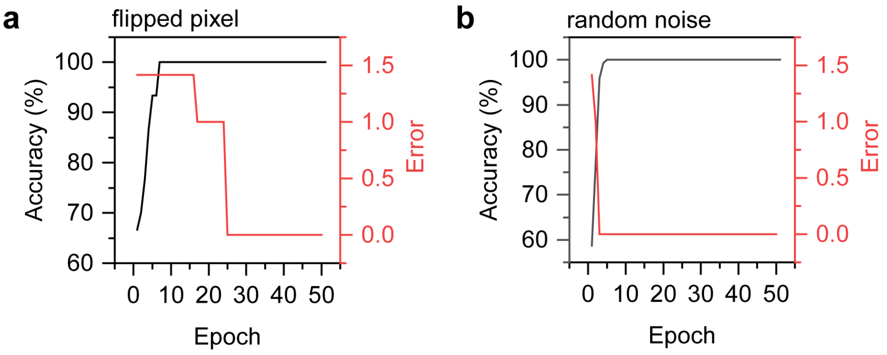

Figure S29. The accuracy and error trends during software spiking neural network (SNN) training for different noises. (a) Flipped pixel noise, (b) random gray scale noise.

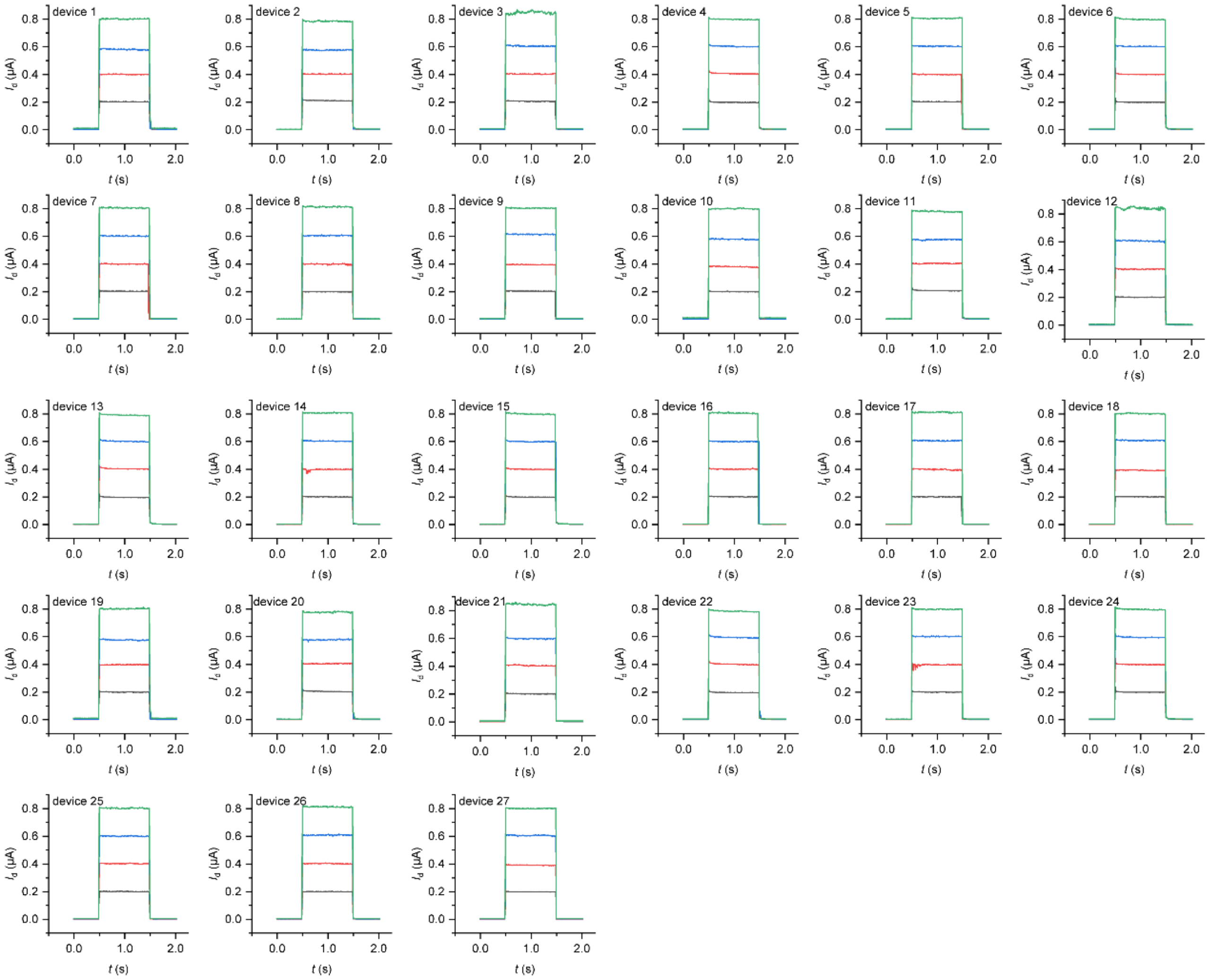

Figure S30. The I_d-t curves of the devices read from a drain pulse (1 V, 1 s). The devices show four levels of conductance by programming.

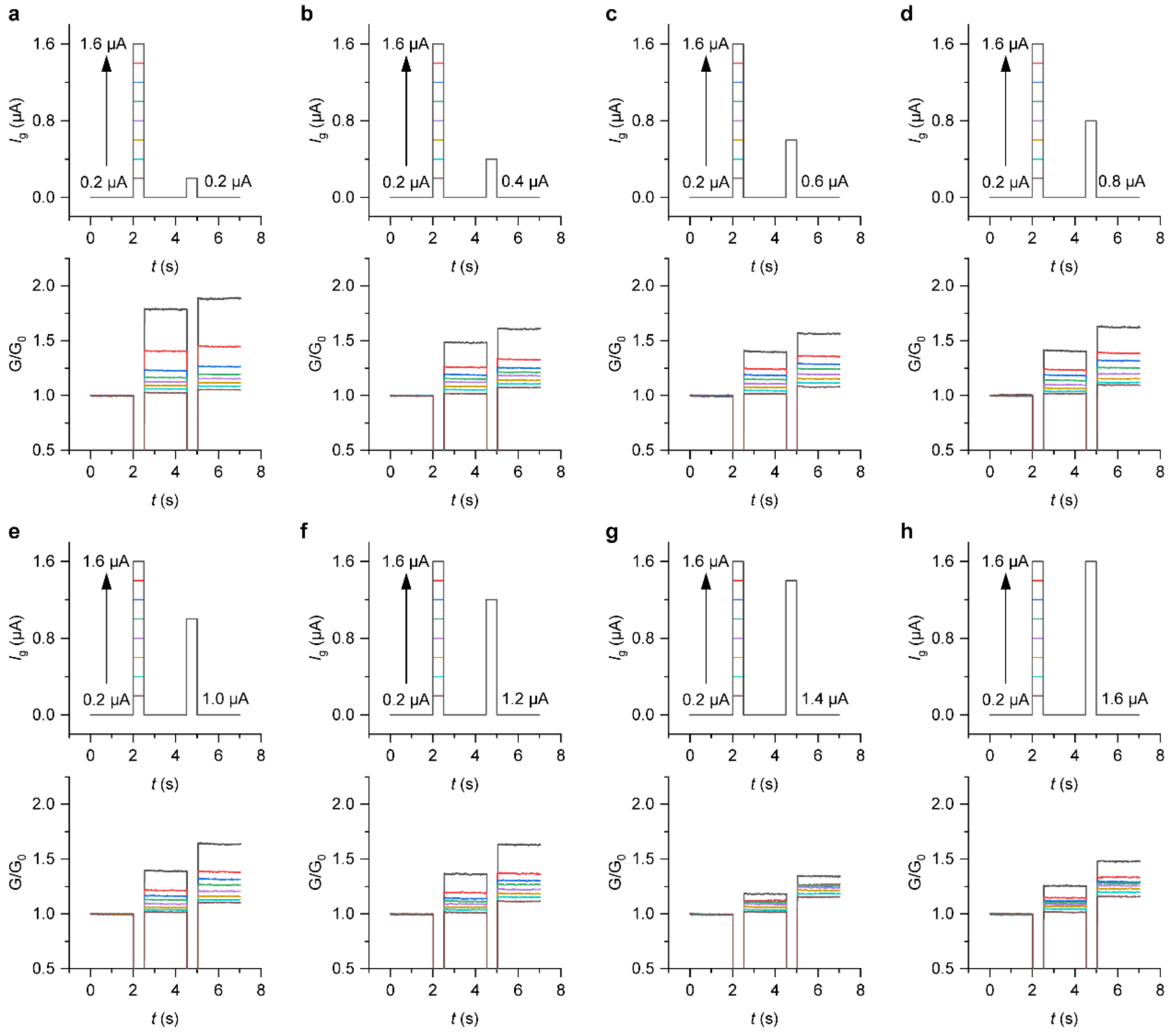

Figure S31. The double gate pulses I_g-t as the programming signals and the corresponding G/G_0-t curves. (a) The first gate pulse amplitude varies from 0.2 μA to 1.6 μA, and the second gate pulse is fixed at 0.2μA. The second gate pulse is fixed from 0.2 μA (a) to 1.6 μA (h), respectively.

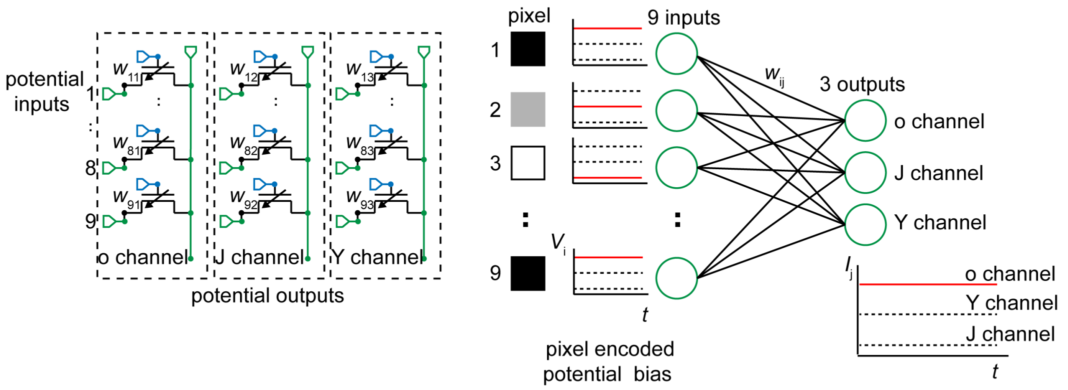

Figure S32. The structure of the artificial neural network. Left: a circuit diagram of an artificial neural network unit. Right: the schematic of the ANN architecture. The image pixels are encoded in various potential

bias as the inputs. The higher grayscale pixels lead to lower potential.

Table S9. The parameters in the flowchart in **Fig. S26**

Symbol	Description
t_{i}	The firing time of the *i*th input neuron
g_{i}	The gray value of the *i*th input neuron
g_{max}	The maximum pixel gray value in input images
t_{max}	The maximum spike time
F_{i}	The spike of the *i*th input in software network
w_{ij}	The synaptic weight in software network from the *i*th input to *j*th output.
P_{j}	The accumulated signal of the *j*th output
S_{j}	The spike of the *j*th output in software network
θ	The threshold to fire a spike of output
t_{j}	Firing time of the *j*th output
T_{j}	The target firing time of the *j*th output
e_{j}	The temporal error function of the *j*th output
L	The loss function
η	The learning rate
F_{iw}	The spike of the *i*th input in hardware network
t_{iw}	The firing time of the *i*th input
t_{p}	The period of one epoch step
P_{jw}	The accumulated signal of the *j*th output in hardware network
w_{ijw}	The normalized weights used in hardware network
G_{j}	The output conductance of the *j*th output in hardware network

Table S10. The parameters and value for the power evaluation

Symbol	value
N_{1st}	9-49
N_{2nd}	3-7
V_{p}	1 V
V_{bias}	0.8-1 V
V_{2nd}	1 V
R_{1st}	1.25-1.67 MΩ
R_{2nd}	0.83 MΩ
t_{pt}	0.001-1.5 s
t_{total}	7 s

Supplementary Reference

1 Liang, X., Luo, Y., Pei, Y., Wang, M. & Liu, C. Multimode transistors and neural networks based on ion-dynamic capacitance. *Nature Electronics* **5**, 859-869 (2022).

2 Brad, A. J. & Faulkner, L. R. *Electrochemical Methods: Fundamentals and Applications*. (John Wiley & Sons, Inc., 2000).

3 Yang, C. S., Shang, D. S., Liu, N., Shi, G., Shen, X., Yu, R. C., Li, Y. Q. & Sun, Y. A Synaptic Transistor based on

Quasi-2D Molybdenum Oxide. *Advanced Materials* **29**, 1700906 (2017).
4 Yang, C. S., Shang, D. S., Liu, N., Fuller, E. J., Agrawal, S., Talin, A. A., Li, Y. Q., Shen, B. G. & Sun, Y. All-Solid-State Synaptic Transistor with Ultralow Conductance for Neuromorphic Computing. *Advanced Functional Materials* **28**, 1804170 (2018).
5 Wang, M., Cai, S., Pan, C., Wang, C., Lian, X., Zhuo, Y., Xu, K., Cao, T., Pan, X., Wang, B., Liang, S.-J., Yang, J. J., Wang, P. & Miao, F. Robust memristors based on layered two-dimensional materials. *Nature Electronics* **1**, 130-136 (2018).
6 Jiang, J., Guo, J., Wan, X., Yang, Y., Xie, H., Niu, D., Yang, J., He, J., Gao, Y. & Wan, Q. 2D MoS2 Neuromorphic Devices for Brain-Like Computational Systems. *Small* **13**, 1700933 (2017).
7 Zhu, X., Li, D., Liang, X. & Lu, W. D. Ionic modulation and ionic coupling effects in MoS2 devices for neuromorphic computing. *Nature Materials* **18**, 141-148 (2018).
8 Wang, B., Wang, X., Wang, E., Li, C., Peng, R., Wu, Y., Xin, Z., Sun, Y., Guo, J., Fan, S., Wang, C., Tang, J. & Liu, K. Monolayer MoS2 Synaptic Transistors for High-Temperature Neuromorphic Applications. *Nano Letters* **21**, 10400-10408 (2021).
9 Yang, J. T., Ge, C., Du, J. Y., Huang, H. Y., He, M., Wang, C., Lu, H. B., Yang, G. Z. & Jin, K. J. Artificial Synapses Emulated by an Electrolyte‐Gated Tungsten‐Oxide Transistor. *Advanced Materials* **30**, 1801548 (2018).
10 Tang, J., Bishop, D. M., Kim, S., Copel, M., Gokmen, T., Todorov, T. K., Shin, S., Lee, K.-T., Solomon, P. M., Chan, K. K. H., Haensch, W. E. & Rozen, J. in *2018 IEEE International Electron Devices Meeting (IEDM).* 13.11.11-13.11.14.
11 Go, J., Kim, Y., Kwak, M., Song, J., Chekol, S. A., Kwon, J.-D. & Hwang, H. W/WO3−x based three-terminal synapse device with linear conductance change and high on/off ratio for neuromorphic application. *Applied Physics Express* **12**, 026503 (2019).
12 Kim, S., Todorov, T., Onen, M., Gokmen, T., Bishop, D., Solomon, P., Lee, K. T., Copel, M., Farmer, D. B., Ott, J. A., Ando, T., Miyazoe, H., Narayanan, V. & Rozen, J. in *2019 IEEE International Electron Devices Meeting (IEDM).* 35.37.31-35.37.34.
13 Lee, J., Nikam, R. D., Lim, S., Kwak, M. & Hwang, H. Excellent synaptic behavior of lithium-based nano-ionic transistor based on optimal WO2.7 stoichiometry with high ion diffusivity. *Nanotechnology* **31**, 235203 (2020).
14 Yao, X., Klyukin, K., Lu, W., Onen, M., Ryu, S., Kim, D., Emond, N., Waluyo, I., Hunt, A., Del Alamo, J. A., Li, J. & Yildiz, B. Protonic solid-state electrochemical synapse for physical neural networks. *Nat Commun* **11**, 3134 (2020).
15 Ge, C., Li, G., Zhou, Q.-l., Du, J.-y., Guo, E.-j., He, M., Wang, C., Yang, G.-z. & Jin, K.-j. Gating-induced reversible HxVO2 phase transformations for neuromorphic computing. *Nano Energy* **67**, 104268 (2020).
16 Li, Y., Lu, J., Shang, D., Liu, Q., Wu, S., Wu, Z., Zhang, X., Yang, J., Wang, Z., Lv, H. & Liu, M. Oxide‐Based Electrolyte‐Gated Transistors for Spatiotemporal Information Processing. *Advanced Materials* **32**, 2003018 (2020).
17 Huang, F., Liu, H., Li, X. & Wang, S. Enhancing hole injection by processing ITO through MoO3 and self-assembled monolayer hybrid modification for solution-processed hole transport layer-free OLEDs. *Chemical Engineering Journal* **427**, 131356 (2022).
18 Meng, X., Ji, W., Hua, J., Yu, Z., Zhang, Y., Li, H. & Zhao, J. Efficient, air-stable quantum dots light-emitting devices with MoO3 modifying the anode. *Journal of Luminescence* **143**, 442-446 (2013).
19 Liang, D., Xue, X., Peng, J. & Ji, W. Perovskite light-emitting diodes with solution-processed MoO3 films as the hole-transport layers. *Journal of Luminescence* **256**, 119621 (2023).
20 Brinkmann, K. O., Becker, T., Zimmermann, F., Kreusel, C., Gahlmann, T., Theisen, M., Haeger, T., Olthof, S.,

Tückmantel, C., Günster, M., Maschwitz, T., Göbelsmann, F., Koch, C., Hertel, D., Caprioglio, P., Peña-Camargo, F., Perdigón-Toro, L., Al-Ashouri, A., Merten, L., Hinderhofer, A., Gomell, L., Zhang, S., Schreiber, F., Albrecht, S., Meerholz, K., Neher, D., Stolterfoht, M. & Riedl, T. Perovskite–organic tandem solar cells with indium oxide interconnect. *Nature* **604**, 280-286 (2022).

21 Vibavakumar, S., Nisha, K. D., Harish, S., Archana, J. & Navaneethan, M. Synergistic effect of MoO3/MoS2 in improving the electrocatalytic performance of counter electrode for enhanced efficiency in dye-sensitized solar cells. *Materials Science in Semiconductor Processing* **161**, 107431 (2023).

22 Schmidt, H., Flügge, H., Winkler, T., Bülow, T., Riedl, T. & Kowalsky, W. Efficient semitransparent inverted organic solar cells with indium tin oxide top electrode. *Applied Physics Letters* **94**, 243302 (2009).

23 Kishi, M., Kubo, Y., Ishikawa, R., Shirai, H. & Ueno, K. Efficient Organic Photovoltaic Cells Using MoO3 Hole-Transporting Layers Prepared by Simple Spin-Cast of Its Dispersion Solution in Methanol. *Japanese Journal of Applied Physics* **52**, 020202 (2013).

24 Lee, D. J., Kumar, G. M., Kim, Y., Yang, W., Kim, D. Y., Kang, T. W. & Ilanchezhiyan, P. Hybrid CsPbBr3 quantum dots decorated two dimensional MoO3 nanosheets photodetectors with enhanced performance. *Journal of Materials Research and Technology* **18**, 4946-4955 (2022).

25 Bai, Y., Liu, X., Chen, L., Khizar ul, H., Khan, M. A., Zhu, W. Q., Jiang, X. Y. & Zhang, Z. L. Organic thin-film field-effect transistors with MoO3/Al electrode and OTS/SiO2 bilayer gate insulator. *Microelectronics Journal* **38**, 1185-1190 (2007).

26 Zhang, Q., Ou, Q., Hu, G., Liu, J., Dai, Z., Fuhrer, M. S., Bao, Q. & Qiu, C.-W. Hybridized Hyperbolic Surface Phonon Polaritons at α-MoO3 and Polar Dielectric Interfaces. *Nano Letters* **21**, 3112-3119 (2021).

27 Zhang, L., Jin, Z. & Tsubaki, N. Activating and optimizing the MoS2@MoO3 S-scheme heterojunction catalyst through interface engineering to form a sulfur-rich surface for photocatalyst hydrogen evolution. *Chemical Engineering Journal* **438**, 135238 (2022).

28 Smith, R. L. & Rohrer, G. S. The Protonation of MoO3during the Partial Oxidation of Alcohols. *Journal of Catalysis* **173**, 219-228 (1998).

29 Ikeda, Y., Estes, D. P. & Grabowski, B. Comprehensive Understanding of H Adsorption on MoO3 from Systematic Ab Initio Simulations. *The Journal of Physical Chemistry C* **126**, 7728-7738 (2022).

30 Grimme, S., Antony, J., Ehrlich, S. & Krieg, H. A consistent and accurate ab initio parametrization of density functional dispersion correction (DFT-D) for the 94 elements H-Pu. *The Journal of Chemical Physics* **132** (2010).

31 Sha, X., Chen, L., Cooper, A. C., Pez, G. P. & Cheng, H. Hydrogen Absorption and Diffusion in Bulk α-MoO3. *Journal of Physical Chemistry C* **113**, 11399-11407 (2009).

32 Chen, M., Friend, C. M. & Kaxiras, E. The Chemical Nature of Surface Point Defects on MoO3(010): Adsorption of Hydrogen and Methyl. *Journal of the American Chemical Society* **123**, 2224-2230 (2001).

33 Adams, S., Ehses, K.-H. & Spilker, J. Proton ordering in the Peierls-distorted hydrogen molybdenum bronze H0.33MoO3: structure and physical properties. *Acta Crystallographica Section B* **49**, 958-967 (1993).
